# Size-fractionated N_2_ fixation off the Changjiang Estuary during summer

**DOI:** 10.3389/fmicb.2023.1189410

**Published:** 2023-05-09

**Authors:** Zhibing Jiang, Yuanli Zhu, Zhenhao Sun, Hongchang Zhai, Feng Zhou, Xiaojun Yan, Quanzhen Chen, Jianfang Chen, Jiangning Zeng

**Affiliations:** ^1^Key Laboratory of Marine Ecosystem Dynamics, Second Institute of Oceanography, Ministry of Natural Resources, Hangzhou, China; ^2^Key Laboratory of Ocean Space Resource Management Technology, Ministry of Natural Resource, Hangzhou, China; ^3^Key Laboratory of Nearshore Engineering Environment and Ecological Security of Zhejiang Province, Hangzhou, China; ^4^State Key Laboratory of Satellite Ocean Environment Dynamics, Second Institute of Oceanography, Ministry of Natural Resources, Hangzhou, China; ^5^Observation and Research Station of Marine Ecosystem in the Yangtze River Delta, Ministry of Natural Resources, Hangzhou, China; ^6^Marine Science and Technology College, Zhejiang Ocean University, Zhoushan, China

**Keywords:** N_2_ fixation, *Trichodesmium*, unicellular diazotrophs, Changjiang Estuary, Changjiang Diluted Water, Kuroshio

## Abstract

Recent evidence has shown active N_2_ fixation in coastal eutrophic waters, yet the rate and controlling factors remain poorly understood, particularly in large estuaries. The Changjiang Estuary (CE) and adjacent shelf are characterized by fresh, nitrogen-replete Changjiang Diluted Water (CDW) and saline, nitrogen-depletion intruded Kuroshio water (Taiwan Warm Current and nearshore Kuroshio Branch Current), where N_2_ fixation may be contributed by different groups (i.e., *Trichodesmium* and heterotrophic diazotrophs). Here, for the first time, we provide direct measurement of size-fractionated N_2_ fixation rates (NFRs) off the CE during summer 2014 using the ^15^N_2_ bubble tracer method. The results demonstrated considerable spatial variations (southern > northern; offshore > inshore) in surface and depth-integrated NFRs, averaging 0.83 nmol N L^−1^ d^−1^ and 24.3 μmol N m^−2^ d^−1^, respectively. The highest bulk NFR (99.9 μmol N m^−2^ d^−1^; mostly contributed by >10 μm fraction) occurred in the southeastern East China Sea, where suffered from strong intrusion of the Kuroshio water characterized by low N/P ratio (<10) and abundant *Trichodesmium* (up to 10.23 × 10^6^ trichomes m^−2^). However, low NFR (mostly contributed by <10 μm fraction) was detected in the CE controlled by the CDW, where NO_x_ concentration (up to 80 μmol L^−1^) and N/P ratio (>100) were high and *Trichodesmium* abundance was low. The >10 μm fraction accounted for 60% of depth-integrated bulk NFR over the CE and adjacent shelf. We speculated that the present NFR of >10 μm fraction was mostly supported by *Trichodesmium*. Spearman rank correlation indicated that the NFR was significantly positively correlated with *Trichodesmium* abundance, salinity, temperature and Secchi depth, but was negatively with turbidity, N/P ratio, NO_x_, and chlorophyll *a* concentration. Our study suggests that distribution and size structure of N_2_ fixation off the CE are largely regulated by water mass (intruded Kuroshio water and CDW) movement and associated diazotrophs (particularly *Trichodesmium*) and nutrient conditions.

## 1. Introduction

Diazotrophs convert N_2_ into bioavailable ammonia (NH_3_) catalyzed by the enzyme nitrogenase (*nifH*), which substantially relieves nitrogen limitation for primary production over most of the surface ocean (Zehr, 2011). N_2_ fixation is an important new bioavailable nitrogen source and thereby is crucial to marine nitrogen biogeochemical cycle and biological pump function (Falkowski, 1997; Capone et al., 2008). Diazotrophs in marine environment include filamentous cyanobacteria (*Trichodesmium* and diatom-diazotroph associations [DDAs]), unicellular cyanobacteria (UCYN-A, -B, and -C), bacteria, and archaea. *Trichodesmium* and DDAs (*Richelia*/*Calothrix*) are conventionally recognized as the major contributors to marine N_2_ fixation (Capone et al., 1997; Sohm and Webb, 2011; Karl et al., 2012). However, Zehr et al. (2001) reported that N_2_ fixation rate (NFR) of unicellular cyanobacteria can equal or exceed that of *Trichodesmium* at station ALOHA in subtropical North Pacific. Thereafter, accumulating evidence during the last two decades have shown unicellular cyanobacteria and noncyanobacterial diazotrophs (particularly proteobacteria) to be more widespread and actively N_2_ fixing in marine environments than previously thought (Zehr et al., 2001; Montoya et al., 2004; Moisander et al., 2010; Turk-Kubo et al., 2022).

Unicellular diazotrophs fix N_2_ not only in warm ocean, but also in low-temperature (<20°C) temperate, subpolar and polar open seas (Moisander et al., 2010; Shiozaki et al., 2020; Messer et al., 2021; Turk-Kubo et al., 2022). Furthermore, active N_2_ fixation of unicellular diazotrophs was observed in estuaries (Bentzon-Tilia et al., 2014), oxygen minimum zones (Hamersley et al., 2011), and even eutrophic coastal waters (Li et al., 2019, 2020) and upwelling (Wen et al., 2017). Because of light requirement, *Trichodesmium* and DDAs fix N_2_ limited in the euphotic zone during daylight that highly associated with photosynthesis (Breitbarth et al., 2008; Zhu et al., 2022). Unlike them, unicellular diazotrophs, particularly noncyanobacterial diazotrophs, are thought to fix N_2_ utilized organic carbon in the cold, twilight zone and even dark conditions other than in the euphotic zone (Montoya et al., 2004; Bentzon-Tilia et al., 2014; Turk-Kubo et al., 2022). Recent studies revealed more contribution of unicellular diazotrophs to N_2_ fixation in some regions characterized by depleted dissolved iron (dFe), such as the South China Sea (Chen et al., 2013; Wu et al., 2018), tropical and subtropical offshore Pacific (Bonnet et al., 2009, 2018; Kitajima et al., 2009; Shiozaki et al., 2014b; Zhang et al., 2019), and eastern Indian Ocean (Shiozaki et al., 2014a; Wu et al., 2022), because of the higher requirement and acquisition for Fe in *nifH* and photosynthetic enzymes complex of *Trichodesmium* compared to unicellular cyanobacteria (Berman-Frank et al., 2007). According to this ecological trade-offs, unicellular diazotrophs usually dominate under dFe-depletion conditions relative to *Trichodesmium* (Sohm and Webb, 2011; Bonnet et al., 2018; Wen et al., 2022). Additionally, heterotrophic diazotrophs likely dominate the diazotrophic community and actively fix N_2_ in coastal eutrophic waters (Bentzon-Tilia et al., 2014; Li et al., 2019, 2020), which may be facilitated by fresh bioavailable dissolved organic carbon from non-diazotrophic phytoplankton (Li et al., 2019). Therefore, the unicellular diazotrophs greatly broaden the N_2_ fixation domain worldwide and can support a significant fraction of nitrogen budget (Zehr et al., 2001; Montoya et al., 2004; Moisander et al., 2010).

Exploring composition and NFR of diazotrophs will deepen the understanding in contributors and flux of N_2_ fixation. However, direct measurement of NFR in coastal waters, particularly in large estuaries, contributed by filamentous cyanobacteria and unicellular diazotrophs is still lacking. Size-fractionated ^15^N_2_ tracer incubations for N_2_ fixation are widely adopted to roughly distinguish the contribution of large-sized (>10 μm) filamentous cyanobacteria (mainly *Trichodesmium* and DDAs) and small-sized (<10 μm) unicellular diazotrophs (mainly UCYN-A, -B, -C and proteobacteria), despite the controversy (Bonnet et al., 2009; Benavides et al., 2016). The incubation results of size-fractionated NFRs substantially confirm their niche-specific adaptation to different nutrient conditions, such as dFe-replete Kuroshio (Chen et al., 2013), Solomon Sea (Bonnet et al., 2015) and Melanesian archipelago waters (Bonnet et al., 2018) and dFe-depleted Coral Sea (Bonnet et al., 2015), South China Sea (Chen et al., 2013), and South Pacific gyre (Bonnet et al., 2018).

**Figure 1:**
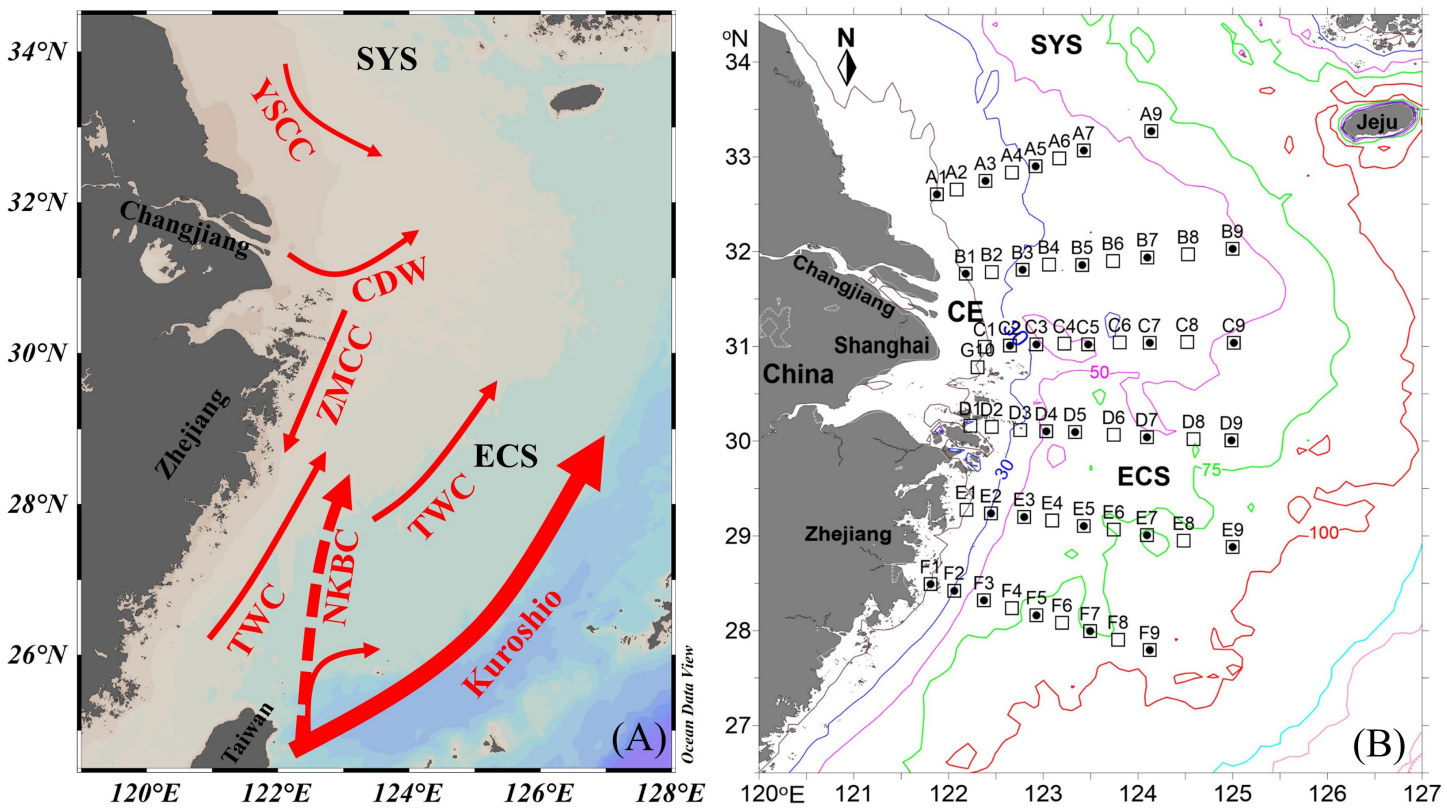
**(A)** Circulation pattern and **(B)** sampling stations off the Changjiang Estuary (CE) during summer. Solid circle, N_2_ fixation rate (NFR) measured stations; ECS, east China sea; SYS, southern yellow sea; CDW, Changjiang Diluted Water; ZMCC, Zhemin coastal current; YSCC, yellow sea coastal current; TWC, Taiwan warm current; NKBC: nearshore Kuroshio branch current.

The Changjiang Estuary (CE) and adjacent East China Sea (ECS) and southern Yellow Sea are primarily characterized by eutrophic, low-salinity Changjiang Diluted Water (CDW) and oligotrophic, warm, saline nearshore Kuroshio Branch Current (NKBC; modified Kuroshio subsurface water) and Taiwan Warm Current (TWC; a mixture of the intruded Kuroshio water and the Taiwan Strait water) (Figure 1A). Although nitrogen repletion and low temperature (<20°C) in the CE restrain the growth of *Trichodesmium* and N_2_ fixation (Zhang et al., 2012; Jiang et al., 2018, 2023), relatively high NFR (3.37 nmol N L^−1^ d^−1^) was observed during spring (Lin et al., 2013), likely contributed by unicellular diazotrophs, particularly heterotrophic diazotrophs. Conversely, abundant *Trichodesmium* (Jiang et al., 2018, 2019) and extremely high NFR (up to 62 nmol N L^−1^ d^−1^; Zhang et al., 2012; Shiozaki et al., 2015; Wu et al., 2018) occurred in the Kuroshio, which largely enhanced N_2_ fixation in the ECS (Jiang et al., 2023). These results showed that NFR in the ECS was highly associated with *Trichodesmium*, probably indicating a high contribution of *Trichodesmium* to N_2_ fixation. The contribution of filamentous cyanobacteria to N_2_ fixation in the ECS during summer was conservatively estimated to be >60% according to their densities in euphotic zone (Jiang et al., 2023). However, Wu et al. (2018) found that unicellular diazotrophs contributed more N_2_ fixation than *Trichodesmium* at two stations near the ECS Kuroshio using the acetylene reduction assay. This controversy warrants further direct measurement of size-fractionated NFR in the CE and adjacent ECS.

To address this gap, size-fractionated incubations for N_2_ fixation were conducted off the CE during summer 2014 using the ^15^N_2_ bubble method. *Trichodesmium* abundance and physicochemical properties were also determined synchronously. For the first time, we provide data on size-fractionated NFRs off the CE, which is useful for understanding relative contribution of filamentous and unicellular diazotrophs to N_2_ fixation and the driving forces.

## 2. Materials and methods

### 2.1. Cruises and sampling stations

A cruise on the CE and adjacent shelf was conducted aboard the R/V *Zhehaihuanjian* during August 2014. A total of 6 transects (A–F) and 53 stations were set (Figure 1B). NFRs were measured at 4–5 stations along each transect.

### 2.2. Sample collection and analysis

Vertical profiles of temperature, salinity, density (σ_t_), turbidity, and depth were measured *in situ* at all stations applying a SBE 917 Plus CTD recorder. The stratification index of the water column was defined as Δσ*_t_* = bottom density − surface density (Huang and Russell, 1994). Secchi depth was determined using a Secchi disk. Seawater samples from 2 to 7 depths (2, 10, 20, 30, 50, 75 m, bottom) at each station (<100 m) were collected by 12-L Niskin bottles mounted on a rosette sampler for analysis of nutrients, nitrogen isotopes, chlorophyll *a*, and *Trichodesmium*. NO_x_ (NO_3_^−^ + NO_2_^−^) and dissolved inorganic phosphorus (DIP) were measured using a continuous-flow analyzer (Skalar San^++^). Water samples (100 mL) for chlorophyll *a* analysis were filtered onto 0.7-μm GF/F filters using low vacuum pressure. After extraction in 90% acetone for 24 h at −20°C, chlorophyll *a* concentrations were determined using a Turner Design Fluorometer. *Trichodesmium* samples were also collected vertically from the bottom to the surface by using a 76-μm mesh net (0.1 m^2^ in mouth acreage) at a tow speed of 0.5 m s^−1^. The net was fitted with a digital flow meter (model 438,115, Hydro-Bios, Germany) to estimate the total volume of seawater passing through it. All net-and water-collected samples were preserved in 4% formalin, and the sedimentation and concentration processes used are described in Jiang et al. (2017, 2018). To sink the trichomes, glacial acetic acid was added (final concentration of 1%) to collapse the gas vesicles in the *Trichodesmium* cells before the sedimentation process (Detoni et al., 2016; Jiang et al., 2018). Colonial and free trichomes of *Trichodesmium* were enumerated using a Leica DMI3000B fluorescence microscope.

### 2.3. Incubation and measurement N_2_ fixation rate (NFR)

The NFR was measured using the ^15^N_2_ bubble method (Montoya et al., 1996; Zhang et al., 2012) rather than the dissolution method (Mohr et al., 2010), because of the time limitation and extremely high environmental (e.g., salinity, turbidity, and nutrients) gradients among stations and layers under domination of upper fresh, eutrophic CDW and deeper saline, oligotrophic NKBC, and TWC water. According to the PAR in the water column, seawater for the NFR incubation was collected from 5 depths that corresponded to 100, 50, 33, 10, and 1% of surface irradiance using Niskin bottles. Duplicate water samples were filled bubble free into 580-mL transparent glass bottles. After filling, 1 ml of 99 at% ^15^N_2_ (Cambridge Isotope Laboratories) was spiked with a septum using an Agilent gastight syringe, with the pressure across the septum balanced by another syringe. To facilitate the equilibration of the ^15^N_2_ gas bubble, each bottle was gently shaken for 3 min before incubation. Incubations were placed in deck-board incubators covered with neutral-density screens to adjust light densities (100, 50, 33, 10, and 1% of natural sea-surface irradiance) and cooled with continuously circulating surface seawater. After 24 h, each incubated sample was prefiltered through a 10-μm pore size Millipore nylon membrane (47 mm diameter) and then by a precombusted (4 h at 450°C) GF/F filter (25 mm diameter). The particles collected on the GF/F filters were of the <10 μm fraction. The particles collected on the Millipore membranes were transferred to another GF/F filters by repeated washing and rinsing with deionized water and thus were of the >10 μm fraction (Zhang et al., 2019). However, few particles might retain on the Millipore membranes, resulting in potential error in measuring NFRs of bulk and > 10 μm fraction. These filter samples were immediately stored at −20°C for further analysis. Natural ^15^N abundance (‰) in particulate organic nitrogen (PON) was measured for calculating the ^15^N enrichment during incubation. The filters for PON and ^15^N measurements were dried at 60°C and pelletized in tin capsules. The PON concentration and isotopic ratios of ^15^N:^14^N were measured using a Flash 2000 elemental analyzer coupled to a Thermo Finnigan Delta Plus isotope ratio mass spectrometer. The reproducibility of δ^15^N-PON measurements was typically better than 0.2‰. The NFRs and their detection limits (minimum quantifiable rates; 0.03–0.70 nmol N L^−1^ d^−1^) were calculated following Montoya et al. (1996). The bulk NFR was obtained by adding NFRs of >10 μm and < 10 μm fractions. Depth-integrated NFR at each station was calculated by trapezoidal integration over the sampling depths in the euphotic zone.

### 2.4. Data analysis and statistical tests

Depth-integrated densities (DIDs) of their trichomes were estimated by trapezoidal integration over the sampling depths. Net-collected samples are prone to result in an underestimation of the *Trichodesmium* density due to the easy loss of free trichomes, while colonies can be easily missed in water-collected samples because of the very limited volume (1 l). Therefore, we corrected total DID of *Trichodesmium* using water-collected DID of free trichomes and net-collected DID of colonial trichomes (Jiang et al., 2018). Spearman rank correlation or regression analysis between NFRs (bulk and >10 μm fractions) and environmental variables and *Trichodesmium* abundance was performed using SPSS 20.0. Figures depicting the distribution of environmental variables, *Trichodesmium* density, and NFR were constructed using ODV 4.

## 3. Results

### 3.1. Physicochemical conditions

**Figure 2:**
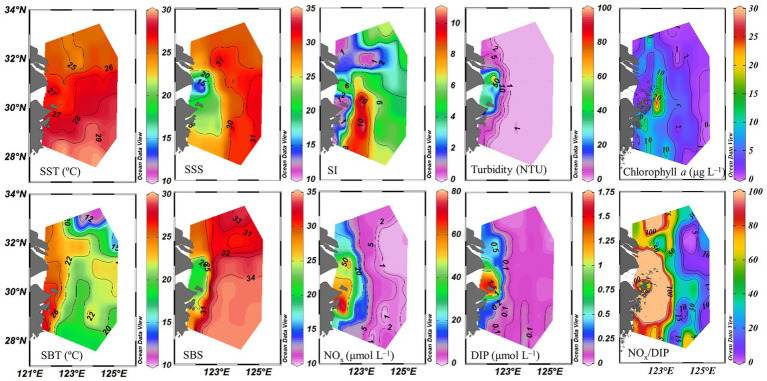
Distribution of physicochemical parameters off the CE. SST, sea surface temperature; SBT, sea bottom temperature; SSS, sea surface salinity; SBS, sea bottom salinity; SI, stratification index; NO_x_, (NO_3_^−^ + NO_2_^−^); DIP, dissolved inorganic phosphorus.

**Figure 3:**
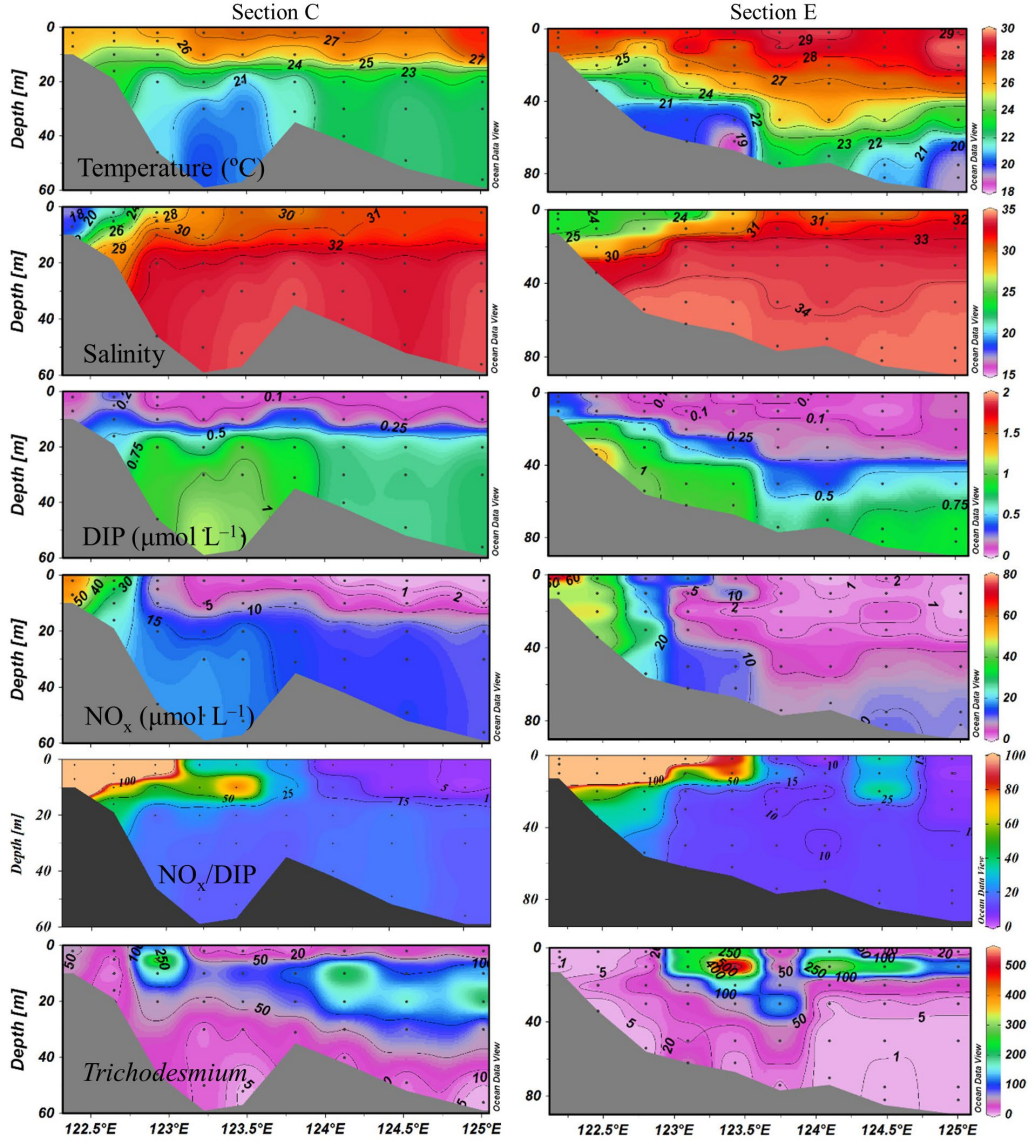
Vertical profiles of physicochemical parameters and *Trichodesmium* abundances (trichomes L^−1^) along transects C and E.

Sea surface temperature ranged from 24.3 to 29.5°C. The eutrophic CDW extended southeasterly, resulting in low salinity (<31) and high turbidity (>10 NTU), concentrations of NO_x_ (>20 μmol L^−1^), DIP (>0.5 μmol L^−1^) and chlorophyll *a*, and NO_x_/DIP ratio (>100) on the surface in the CE and Zhejiang coastal waters (Figure 2). The isohaline 31 on the surface crossed 32°N and the isohaline 33 on the bottom approached to 32°N, indicating strong intrusions of the NKBC and TWC water into the northern part of the CE. High stratification (with stratification index >10 kg m^−3^) was observed in Zhejiang coastal waters, because of upper warm, low-salinity CDW and deeper cold, high-salinity Kuroshio intrusion water. Relatively abundant DIP (up to 0.1 μmol L^−1^) and low NO_x_/DIP ratio (<10) were observed in the southeastern ECS along with the Kuroshio intrusion path, indicating a strong nitrogen limitation. Secchi depth ranged from 1.5 to 14.5 m with an average of 8.2 ± 3.6 m, which showed higher water clarity in the southern ECS than in the CE. The surface PAR off the CE was high (>2000 μmol photons m^−2^ s^−1^) at noon on the sunny day. The vertical profiles of temperature and salinity along transects C and E showed upward isotherm and isohaline (Figure 3), indicating a marked upwelling due to strong incursions of the NKBC and TWC. Nitrogen limitation was observed in the subsurface water column, where NO_x_/DIP ratio was low (<15) because of domination of the NKBC and TWC water.

### 3.2. *Trichodesmium* abundance

**Figure 4:**
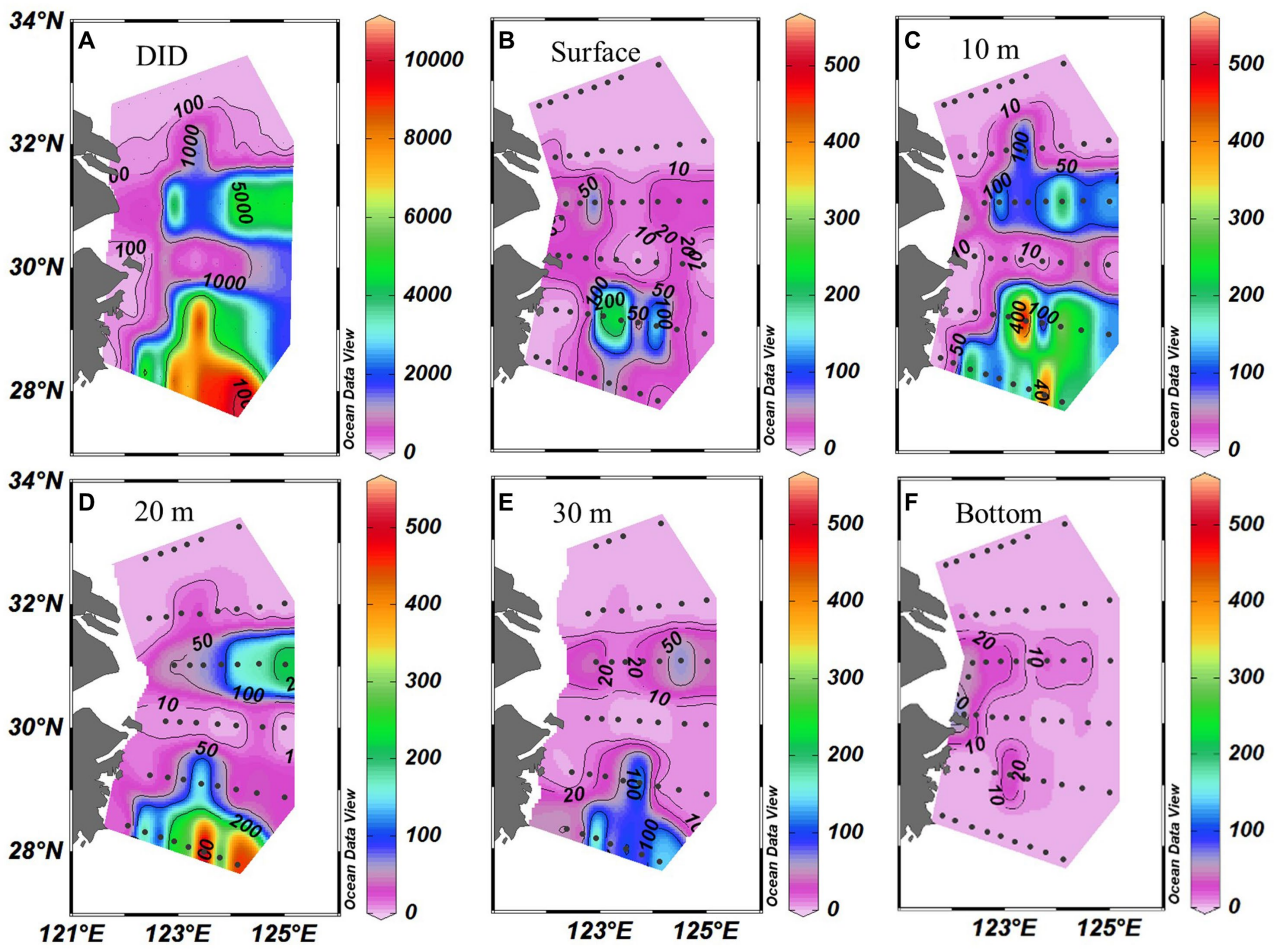
Distribution of *Trichodesmium* **(A)** DIDs (Depth-integrated density; ×10^3^ trichomes m^−2^) and **(B–F)** densities (trichomes L^−1^) at different depths off the CE.

**Figure 5:**
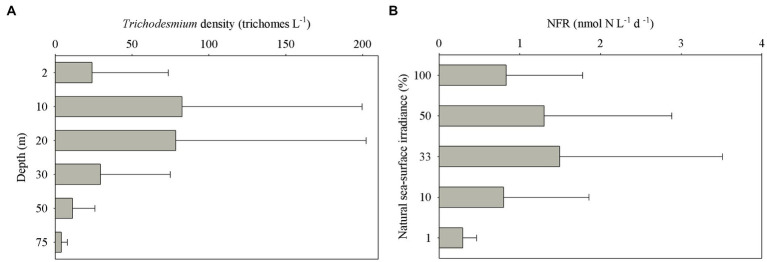
Vertical distribution of **(A)** *Trichodesmium* densities at different depths and **(B)** N_2_ fixation rates (NFRs) at different light intensity off the CE.

*Trichodesmium* was abundant in the southeastern ECS (with the highest DID of 10.23 × 10^6^ trichomes m^−2^) and in the eastern ECS away from the CE (Figure 4). The distribution of *Trichodesmium* abundance in the upper 20 m water column was contrary to the distribution of NO_x_ concentration. Notably, abundant *Trichodesmium* in the 10–30 m water column extended tongue-shapely from the southeastern ECS (Kuroshio) to the CE. Moreover, relative high abundance of *Trichodesmium* was observed on the bottom of the CE. Figure 3 shows obvious subsurface (10–30 m depth) maximum of *Trichodesmium* abundance along transects C and E. Figure 5 confirms that *Trichodesmium* densities were higher at 10, 20, and 30 m depths than on the surface and at 30, 50, and 75 m depths, with average densities of 82.4, 78.4, 29.3, 25.1, 23.9, 11.9, and 3.9 trichomes L^−1^, respectively.

### 3.3. Size-fractionated NFR

**Figure 6:**
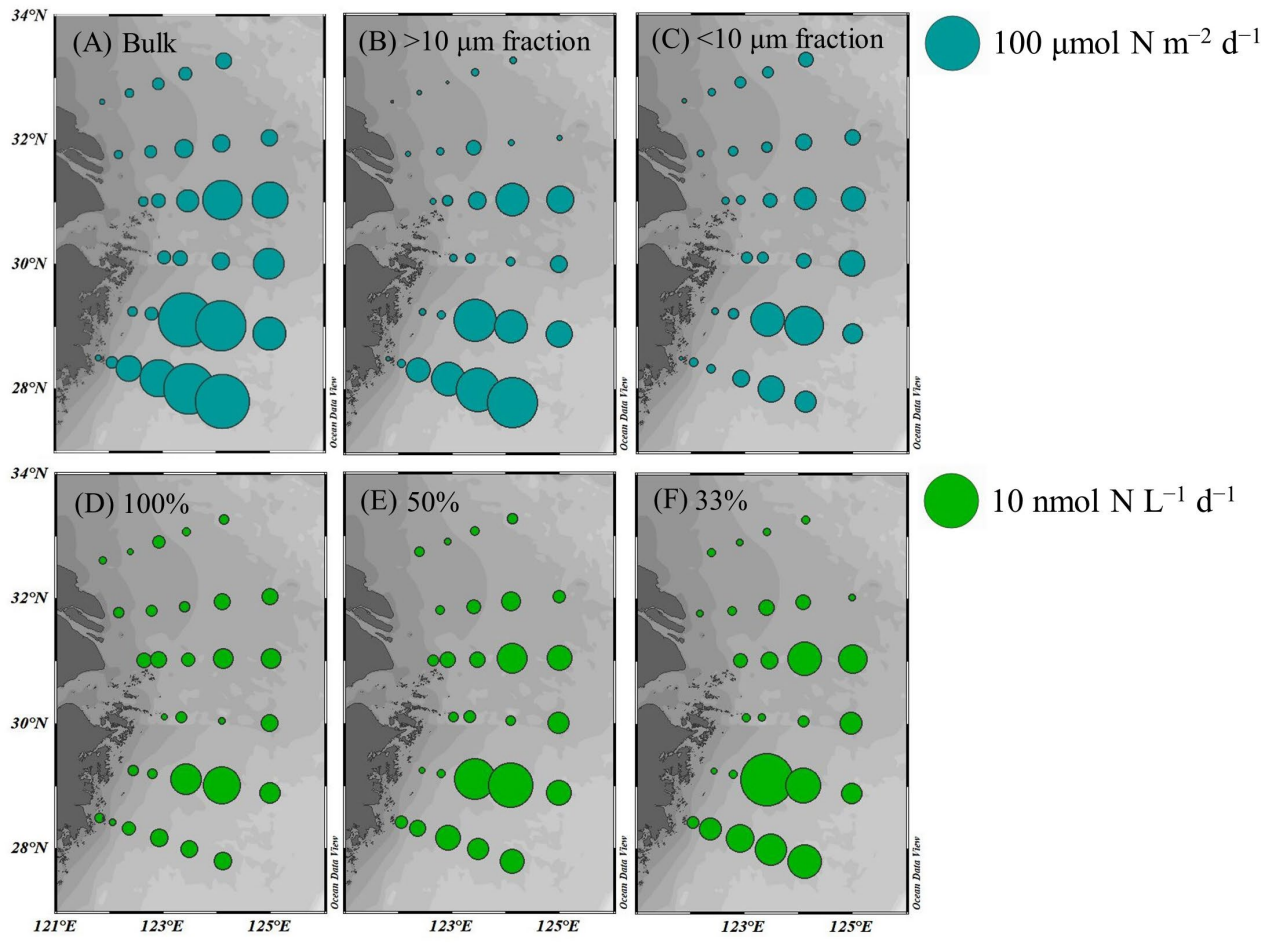
Spatial distribution of NFRs **(A–C)** in the euphotic zone (depth-integrated NFR; μmol N m^−2^ d^−1^) and **(D–F)** at different light intensities (nmol N L^−1^ d^−1^) off the CE. A: bulk NFR; B: NFR of >10 μm fraction; C: NFR of <10 μm fraction; D: 100% surface irradiance; E: 50% surface irradiance; F: 33% surface irradiance.

The average bulk NFR off the CE during summer was 24.3 ± 30.7 μmol N m^−2^ d^−1^. The average NFRs of >10 and < 10 μm fractions were 14.7 ± 22.1 and 9.6 ± 11.2 μmol N m^−2^ d^−1^, respectively, indicating a high contribution (60%) of >10 μm diazotrophs to N_2_ fixation. Figure 6 shows higher NFRs in the offshore and southern parts than in the inshore and northern parts. The highest bulk NFR (99.9 μmol N m^−2^ d^−1^) occurred in the southeastern ECS, which largely contributed by >10 μm fraction. The NFR distribution of >10 μm and <10 μm fractions was consistent with the bulk NFR distribution. The bulk NFRs at 100, 50, 33, 10, and 1% of natural sea-surface irradiance were 0.83, 1.30, 1.50, 0.80, and 0.31 nmol N L^−1^ d^−1^, respectively (Figure 5).

### 3.4. Relationship analysis

**Figure 7:**
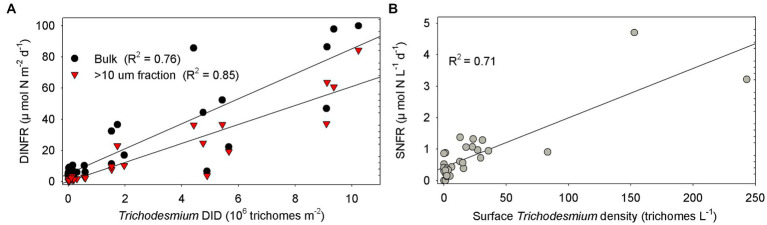
Correlation between NFRs (bulk and > 10 μm fraction) and *Trichodesmium* densities **(A)** in the water column (DID) and **(B)** on the surface off the CE. DINFR: depth-integrated NFR; SNFR: surface NFR.

Spearman rank correlation showed that the surface and depth-integrated NFRs were significantly (*p* < 0.01) positively correlated with temperature, salinity, and Secchi depth, but was negatively with turbidity, NO_x_, and NO_x_/DIP ratio (Table 1). Notably, NFR of >10 μm fraction significantly (*p* < 0.05) positively correlated with stratification index, but non-significant positive correlation between NFR of <10 μm fraction and stratification index was found. The surface and depth-integrated NFRs were significantly (*p* < 0.001) positively correlated with surface density and DID of *Trichodesmium*, respectively. Figure 7 confirms high correlation coefficient (>0.7) between NFRs (particularly >10 μm fraction) and *Trichodesmium* densities.

**Table 1:** Spearman rank correlation between N_2_ fixation rates (NFRs) and physicochemical factors and *Trichodesmium* density off the CE during summer.

Parameters	Bulk DINFR	>10 μm fraction DINFR	<10 μm fraction DINFR	Bulk SNFR
Temperature	0.389^*^	0.527^**^	0.235	0.475^**^
Salinity	0.856^***^	0.797^***^	0.831^***^	0.446^*^
Stratification index	0.242	0.373^*^	0.137	0.078
Secchi depth	0.645^***^	0.424^*^	0.763^***^	0.425^*^
Surface turbidity	−0.556^***^	−0.392^*^	−0.657^***^	−0.403^*^
NO_x_	−0.819^***^	−0.669^***^	−0.825^***^	−0.534^**^
DIP	−0.644^***^	−0.446^*^	−0.643^***^	−0.123
NO_x_/DIP ratio	−0.686^***^	−0.675^***^	−0.726^***^	−0.460^**^
Chlorophyll *a*	−0.438^*^	−0.195	−0.579^***^	−0.289
Surface density of *Trichodesmium*	0.638^***^	0.764^***^	0.485^**^	0.703^***^
DID of *Trichodesmium*	0.806^***^	0.890^***^	0.553^**^	0.670^***^

DINFR, depth-integrated NFR; SNFR: surface SNFR. Note that DINFR correlates with average temperature, salinity, nutrients, and chlorophyll *a* of the upper 30 m water column and SNFR correlates with temperature, salinity, nutrients, and chlorophyll *a* on the surface. ^*^p < 0.05; ^**^p < 0.01; ^***^p < 0.001.

## 4. Discussion

### 4.1. Comparison of size-fractionated NFR

For the first time, we report the direct measurement of size-fractionated NFRs off the CE using ^15^N_2_ isotope tracer. With the development and improvement of isotope ratio mass spectrometry technique, NFRs are currently measured using the ^15^N_2_ isotope tracer method instead of acetylene reduction method during the past two decades, because of the advantages in sensitivity and operability (Montoya et al., 1996; Sohm and Webb, 2011). NFRs in the ECS were almost measured using the ^15^N_2_ isotope tracer method (Zhang et al., 2012; Shiozaki et al., 2015; Jiang et al., 2023), except for Wu et al. (2018) who used the acetylene reduction method. Therefore, our measured data of NFRs off the CE is well comparable to the previous results. Notably, the present NFRs obtained using the ^15^N_2_ bubble method may underestimate due to the insufficient dissolution of ^15^N_2_ gas injected (Mohr et al., 2010; Li et al., 2023). However, the level of underestimation of this method is thought to be low in *Trichodesmium*-dominant waters, because *Trichodesmium* can float to the top of the bottle and directly use the added ^15^N_2_ gas (Großkopf et al., 2012). Additionally, because the equilibration between the injected ^15^N_2_ gas and seawater usually increases over time, the 24-h incubation will likely minimize the artifact of insufficient dissolution of ^15^N_2_ gas (Wannicke et al., 2018; Li et al., 2023). The present NFRs seem unlikely to be overestimated due to ^15^N contamination, since Cambridge ^15^N_2_ gas has been observed to has minimal levels of ^15^N-ammonium contamination equivalent to rates of < 0.02 nmol N L^−1^ d^−1^ that are below our detection limits (Dabundo et al., 2014). The measurement of size-fractionated NFRs has been widely adopted, including the South China Sea, Mediterranean Sea, Southern California Bight, Coral Sea, Kuroshio, western Pacific, and North Atlantic (references shown in Table 2).

**Table 2:** Comparison of size-fractionated surface (nmol N L^−1^ d^−1^) and depth-integrated (μmol N m^−2^ d^−1^) NFRs off the CE and in other global seas.

Region	Time	SNFR	DINFR	Contribution of >10 μm fraction to SNFR	Contribution of >10 μm fraction to DINFR	Method	Reference
Temperate eastern North Atlantic	2009.07–08	0.13	n.d.	15.1%	n.d.	AR	Benavides et al. (2011)
		0.07	n.d.	23.4%	n.d.	GB	Benavides et al. (2011)
Equatorial Pacific	2006.08–10	23.10	133.73^a^	17%	n.d.	GB	Bonnet et al. (2009)
Mediterranean Sea	2008.06–07	0.40	34.38	25–55%^b^	n.d.	GB	Bonnet et al. (2011)
Eastern Mediterranean Sea	2006.06–2007.05	0.4–4.5	n.d.	50–70%	n.d.	AR	Zeev et al. (2008)
Upwelling off NW Iberia	2014.02–2015.12	n.d.	0.1–1.6	n.d.	Up to 100%	GB	Moreira-Coello et al. (2017)
ALOHA of North Pacific	2004.10–2007.10	2.58	111	17%	n.d.	GB	Church et al. (2009)
Tropical and subtropical North Pacific	2004.11–12	12.97	n.d.	28.1%	n.d.	AR	Kitajima et al. (2009)
	2005.06	5.45	n.d.	32.6%	n.d.		
West of 172°W, 17°S, Pacific	2009.04–06	3.72	n.d.	76%	n.d.	GB	Shiozaki et al. (2014b)
146°–172°W, 17°S, Pacific		1.42	n.d.	46%	n.d.	GB	Shiozaki et al. (2014b)
East of 146°W, 17°S, Pacific		0.30	n.d.	27%	n.d.	GB	Shiozaki et al. (2014b)
Benguela upwelling, South Atlantic	2007.12	<8	n.d.	65–80%	n.d.	GB	Sohm et al. (2011)
Subtropical North Atlantic	2011.01–03	6.24	n.d.	61.5%	n.d.	GD	Benavides et al. (2013)
SPOTS of Southern California Bight	2004.07–2006.09	5.8	n.d.	30%	n.d.	GB	Hamersley et al. (2011)
SMBO of Southern California Bight	2006.07–2007.07	2.4	150	12%	n.d.	GB	Hamersley et al. (2011)
37°N–35°S transect, Atlantic	2005.10–11	0.75	34.41	n.d.	53.5%^c^	GB	Moore et al. (2009)
Norther South China Sea	2008–2010	1.2	51.7	49%^d^	n.d.	GB	Chen et al. (2013)
Upstream Kuroshio	2008–2010	4.0	142.7	55%^d^	n.d.		
Arafura and Timor shelves	2012.12	23	n.d.	74%	n.d.	GB	Messer et al. (2016)
	2013.07–08	60	n.d.	5%	n.d.		
Coral Sea	2012.12	13	n.d.	46%	n.d.	GB	Messer et al. (2016)
	2013.07–08	56	n.d.	16%	n.d.		
Spencer Gulf (33°–36°S), South Australia	2014.04–05; 2014.12	2–64	n.d.	~0	n.d.	GB	Messer et al. (2021)
Solomon Sea	2012.07	≤89.5	624	n.d.	65%	GB	Bonnet et al. (2015)
Coral Sea	2012.09	≤2.8	30	n.d.	70%		
Melanesian archipelago waters	2015.02–03	n.d.	631	n.d.	47.1–83.8%^e^	GB^f^	Bonnet et al. (2018)
Eastern Indian Ocean	2018.03–05	0.032–0.189	4.52–30.16	0–63.4%	0–71.2%	GD	Wu et al. (2022)
CE and adjacent shelf	2014.08	0.83	24.3	n.d.	60%	GB	This study

n.d., no data; AR, acetylene reduction; GB, ^15^N_2_ gas bubble; GD, ^15^N_2_ gas dissolution.

^a^ excluding a high NFR of 25,750 μmol N m^−2^ d^−1^; ^b^ > 3 μm; ^c^ > 20 or 10 μm; ^d^ contribution of *Trichodesmium*; ^e^ > 20 μm; ^f^ GB and nanoscale secondary ion mass spectrometry.

The present average surface (0.83 nmol N L^−1^ d^−1^) and depth-integrated (24.3 μmol N m^−2^ d^−1^) NFRs off the CE were comparable to those (0.70 nmol N L^−1^ d^−1^ and 41 μmol N m^−2^ d^−1^) reported in the ECS shelf (Zhang et al., 2012). However, these measured NFRs were lower than our previous measurement (1.54 nmol N L^−1^ d^−1^ and 89.8 μmol N m^−2^ d^−1^) in the ECS (including the Kuroshio mainstream) during summer 2013 (Jiang et al., 2023). The NFRs were also substantially lower those measured in the ECS near Japan (28 nmol N L^−1^ d^−1^ and 170 μmol N m^−2^ d^−1^) and the Kuroshio (5.61 nmol N L^−1^ d^−1^ and 199 μmol N m^−2^ d^−1^) (Shiozaki et al., 2015). These inconsistent results were attributed to the present study that limited in the CE and adjacent shelf, but did not cover the ECS outer shelf and the Kuroshio mainstream where NFRs were high. Earlier measurement demonstrated significantly higher NFR in the Kuroshio than in the ECS coastal waters (Zhang et al., 2012; Jiang et al., 2023).

Our measured NFRs were comparable to those measured in the Mediterranean Sea (Zeev et al., 2008; Bonnet et al., 2011), Bay of Bengal (Saxena et al., 2020), and a meridional transect in Atlantic (Moore et al., 2009), and higher than those reported in upwelling off NW Iberia (Moreira-Coello et al., 2017), temperate eastern North Atlantic (Benavides et al., 2011), and Eastern Indian Ocean (Wu et al., 2022), but lower than those measured in the northern South China Sea (Lu et al., 2018), upstream Kuroshio (Chen et al., 2013), Southern California Bight (Hamersley et al., 2011), Solomon Sea, Coral Sea, Arafura and Timor shelves (Bonnet et al., 2015; Messer et al., 2016), and tropical and subtropical Pacific and Atlantic (Bonnet et al., 2009, 2018; Church et al., 2009; Kitajima et al., 2009; Wen et al., 2022). Table 2 shows that the present contribution (60%) of >10 μm fraction to N_2_ fixation off the CE was comparable to those detected in the Benguela upwelling, upstream Kuroshio, eastern Mediterranean Sea, Arafura and Timor shelves (in December), tropical western South Pacific, and subtropical North Atlantic, but were higher than those measured in the South China Sea, Southern California Bight, Coral Sea, upwelling off NW Iberia, temperature Spencer Gulf of South Australia, temperate eastern North Atlantic, tropical and subtropical North Pacific, and eastern Indian Ocean. These results suggested that N_2_ fixation were largely contributed by >10 μm fraction (presumably *Trichodesmium*; see discussion below) in the subtropical and tropical seas near mainland/islands or suffered from strong dust inputs where dFe was abundant.

### 4.2. Main contributors to N_2_ fixation

Our direct measured size-fractionated NFR indicated that >10 μm fraction accounted for 60% of bulk NFR, suggesting that N_2_ fixation was most likely to be contributed by large-sized filamentous cyanobacteria. It is widely recognized that NFR of >10 μm fraction in warm ocean is generally contributed by *Trichodesmium* and DDAs (Capone et al., 1997; Sohm and Webb, 2011). This contribution was comparable to our conservative estimation (>60%) by filamentous cyanobacteria in the ECS including the Kuroshio mainstream (Jiang et al., 2023), but was inconsistent with a previous direct measurement (<40%) at two stations of the ECS outer shelf near the Kuroshio using the acetylene reduction assay (Wu et al., 2018). This different contribution was attributed to the large difference of *Trichodesmium* abundance between the present and previous observations. Wu et al. (2018) reported that the density of *Trichodesmium* at station PN04 was below 80 and 15 trichomes L^−1^ on the surface and subsurface, respectively, which were much lower than our observed density in the upper 30 m water column (Figure 4). Particularly in the southeastern ECS (near their measured stations), the present highest density of *Trichodesmium* was 524 trichomes L^−1^. Other previous studies found high surface densities (>2000 trichomes L^−1^) of *Trichodesmium* in the ECS outer shelf and Kuroshio mainstream (Shiozaki et al., 2015; Jiang et al., 2018, 2019).

We speculated that *Trichodesmium* was the main contributor to N_2_ fixation away from the CE, particularly in the eastern and southeastern ECS where high NFR was consistent with abundant *Trichodesmium* (Figures 4, 6). Our pervious investigation showed that DDAs usually limited in the Kuroshio mainstream but sparsely occurred off the CE during summer (Jiang et al., 2019). DDAs were also rarely observed in phytoplankton samples obtained synchronously (unpublished data) and previously (Jiang et al., 2015, 2019) from the CE and adjacent shelf. Similarly, DDAs were not detected in the south of Jeju Island of the ECS using *nifH* gene PCR amplification (Shiozaki et al., 2018). Few unicellular diazotrophs might retain on the 10 μm filters associated with >10 μm fraction. For example, the UCYN-B, recognized as *Crocosphaera*, lives freely (Zehr et al., 2001), colonially (Foster et al., 2013), and also in symbiosis with the diatoms (Carpenter and Janson, 2001). The colonial or symbiotic UCYN-B could also be associated with >10 μm fraction. Noncyanobacterial diazotrophs were found to be attached to large-sized particles [references in Turk-Kubo et al. (2022)]. However, previous molecular studies showed that the dominance of unicellular cyanobacteria diazotrophs decreased from the Kuroshio upstream to downstream and even did not occur in the ECS shelf mixed water (Shiozaki et al., 2018; Cheung et al., 2019), suggesting relatively low probability of unicellular cyanobacteria diazotrophs associated with >10 μm fraction and low contribution of them to N_2_ fixation off the CE. Table 1 shows that NFR was significantly (*p* < 0.001) positively correlated with *Trichodesmium* density. High correlation coefficient (*R*^2^ = 0.85) between NFR of >10 μm fraction and *Trichodesmium* density is also found in Figure 7A. These results confirmed that N_2_ fixation away from the CE (particularly southeastern ECS) was largely contributed by *Trichodesmium*.

Figure 6 shows that <10 μm fraction contributed largely (>50%) to bulk NFR in the CE, particularly in the southern part of the southern Yellow Sea (up to 90%). This finding indicated that unicellular diazotrophs were the main contributors to N_2_ fixation therein. Earlier study demonstrated that heterotrophic diazotrophs (Gammaproteobacteria) were dominant in coastal waters of the southern Yellow Sea using real-time PCR and clone library analysis of *nifH* genes (Zhang et al., 2015). Molecular detection of *nifH* gene revealed that unicellular cyanobacteria diazotrophs had low abundance and even did not occur on the ECS shelf, while proteobacteria presented conversely (Shiozaki et al., 2018; Cheung et al., 2019). Our unpublished data of pyrosequencing based on *nifH* gene during summer 2016 confirmed that proteobacteria overwhelmingly dominated the unicellular diazotrophs at all stations in the CE, but unicellular cyanobacteria diazotrophs rarely detected. Similarly, recent high-throughput sequencing showed that proteobacteria and other heterotrophic unicellular diazotrophs were the main N_2_ fixer in eutrophic Daya Bay (Li et al., 2019) and estuaries (Bentzon-Tilia et al., 2014), which occasionally fixed N_2_ at significant rates (>10 nmol N L^−1^ d^−1^). During spring 2009, relatively active N_2_ fixation (3.37 nmol N L^−1^ d^−1^) was detected in the CE, where temperature averaged at 18.1°C (Lin et al., 2013). Because the growth and N_2_ fixation of *Trichodesmium* are dramatically inhibited at low temperature (<20°C; Breitbarth et al., 2007), unicellular diazotrophs appeared to contribute mostly to spring N_2_ fixation in the CE. These findings suggested that N_2_ fixation probably largely contributed by proteobacteria in the CE and adjacent waters except the eastern and southeastern ECS. Therefore, size-fractionated NFR data is useful to roughly distinguish the relative importance of filamentous and unicellular diazotrophs to N_2_ fixation off the CE.

### 4.3. Controlling factors of size-fractionated NFR

Overall, macro-nutrient stoichiometry indicated that nitrogen shifted across the river plume and upwelling from repletion (NO_x_/DIP ratio > 100) in the CE and Zhejiang coast to deficiency (NO_x_/DIP ratio < 10) in offshore waters, particularly in the southeastern ECS controlled by the Kuroshio intrusion water (Figure 2). Consequently, NFR was high (up to 100 μmol N m^−2^ d^−1^) in the nitrogen-depleted southeastern ECS, but was low (<6 μmol N m^−2^ d^−1^) in the nitrogen-replete CE and Zhejiang coast (Figure 6). This spatial distribution pattern was consistent with previous reports (Zhang et al., 2012; Jiang et al., 2023), which was closely correlated with water mass movement and associated physical properties (e.g., temperature, salinity and turbidity) and *Trichodesmium* density, in addition to NO_x_/DIP. During summer, the shoreward, northward intrusions of oligotrophic, saline TWC, and NKBC strengthen under prevailing southwestern monsoon, which transports abundant *Trichodesmium* off the CE (Jiang et al., 2017, 2018, 2019). However, eutrophic CDW enhances and dominates the CE and strong upwelling occurs along Zhejiang coast, where NO_x_ concentration (up to 80 μmol L^−1^) and NO_x_/DIP ratio (>100) were extremely high (Figures 2, 3). Additionally, relatively abundant dFe (0.47–10.01 nmol L^−1^) was observed in the offshore ECS, because of riverine/atmospheric inputs as well as intrusion of NKBC (Zhang et al., 2022). Figures 4, 6 show abundant *Trichodesmium* and relatively high NFR in the southeastern ECS along the NKBC intrusion, but low *Trichodesmium* density and NFR in the CE characterized by high NO_x_ concentration, NO_x_/DIP ratio and turbidity. Table 1 confirms that NFRs (bulk and > 10 μm fraction) were significantly positively correlated with temperature, salinity, Secchi depth, and *Trichodesmium* abundance, but was negatively with NO_x_ concentration, NO_x_/DIP ratio, and turbidity. Regression analysis showed high correlation between NFRs (particularly >10 μm fraction) and *Trichodesmium* densities (Figure 7). Remarkably, circulation pattern and associated marco-and micro-nutrient conditions profoundly influence N_2_ fixation off the CE. Our previous studies demonstrated that the Kuroshio intrusion significantly enhanced *Trichodesmium* abundance and N_2_ fixation in the ECS through providing appropriate nutrient (N deficiency, abundant DIP and available dFe) environments (Jiang et al., 2018, 2019, 2023). These results suggested that spatial variation in NFR off the CE are largely regulated by *Trichodesmium* abundance and nutrient conditions (particularly NO_x_/DIP ratio) under movement of the intruded Kuroshio water and CDW.

The subsurface (~5–20 m depth) NFRs under 33% (1.50 nmol N L^−1^ d^−1^) and 50% (1.30 nmol N L^−1^ d^−1^) of surface irradiance were much higher than those on the surface and in the deeper water column (Figure 5), highly corresponding to the vertical distribution of *Trichodesmium* abundance (Figure 3). Similar results of measured NFRs were usually observed in the ECS (Zhang et al., 2012; Shiozaki et al., 2015) and other seas (Shiozaki et al., 2018; Wu et al., 2022). These findings suggested that the growth and N_2_ fixation of diazotrophs on the surface off the CE were limited by extremely high solar irradiance (>2000 μmol photons m^−2^ s^−1^ on the sunny day) during summer, while PAR (100–250 μmol photons m^−2^ s^−1^) on the subsurface was favored by diazotrophs. For example, both laboratory and field experiments demonstrated that growth and N_2_ fixation of *Trichodesmium* were light saturated at ~200 μmol quanta m^−2^ s^−1^ (Breitbarth et al., 2008; Lu et al., 2018). In addition, the water mass movement and physicochemical conditions were suitable to growth, accumulation and N_2_ fixation of diazotrophs in the subsurface. The upper nitrogen-replete CDW and deeper nitrogen-depletion, P-replete TWC (modified Kuroshio subsurface water), and NKBC water dominated the CE and adjacent shelf, where subsurface water column was characterized by low concentration of NO_x_ and high concentration of DIP (low NO_x_/DIP ratio) and appropriate temperature and salinity (Figure 3). Earlier studies found that abundant *Trichodesmium* off the CE was transported by the Kuroshio and TWC intrusions, which shaped spatial distribution and N_2_ fixation of filamentous diazotrophs (Shiozaki et al., 2015; Jiang et al., 2017, 2018, 2023). Moreover, NFR of >10 μm fraction was significantly positively correlated with stratification index (Table 1), indicating that *Trichodesmium* benefited from this strong halocline. Therefore, the vertical distribution of N_2_ fixation off the CE was regulated by *Trichodesmium*, irradiance, nutrients and stratification.

Notably, measurable NFRs were detected in the CE (controlled by the eutrophic CDW) and Zhejiang coastal waters (also characterized by strong upwelling) despite high NO_x_ concentration and NO_x_/DIP ratio (Figures 2, 6). Several studies have also observed active N_2_ fixation in coastal eutrophic waters, including estuaries (Bentzon-Tilia et al., 2014), bays (Li et al., 2019, 2020), and upwelling (Wen et al., 2017). Li et al. (2019) hypothesized that active N_2_ fixation (with the maximum NFR of 4.51 nmol N L^−1^ h^−1^) in Daya Bay was facilitated by fresh bioavailable dissolved organic carbon from non-diazotrophic phytoplankton. Algal bloom with high concentration of chlorophyll *a* (~10 μg L^−1^) in the CE might supply abundant fresh dissolved organic carbon for heterotrophic diazotrophs and thereby was conducive to N_2_ fixation. Changjiang inputs huge freshwater and associated nutrients, which forms massive amount of nitrogen-replete CDW, resulting in relatively low NFR (<0.5 nmol N L^−1^ d^−1^) in the CE (Figure 6). From 1980s to 2000s, dissolved inorganic nitrogen and N/P ratio in the CE have increased significantly with exacerbating land-use change and urbanization (Jiang et al., 2014; Zhang et al., 2020). However, nitrogen flux from Changjiang reduced and dissolved inorganic nitrogen concentration and N/P ratio in the CE decreased since 2010, because of comprehensive environmental protection and improvement of Changjiang (Wang et al., 2020). In addition, surface warming and associated stratification enhancement are conducive to growth of *Trichodesmium* (Table 1; Jiang et al., 2018). We expect that N_2_ fixation will contribute more importantly to nitrogen budget and biogeochemical processes off the CE in the future.

## 5. Conclusion

The present study provides the first direct measurement of size-fractionated NFRs off the CE during summer using the ^15^N_2_ bubble tracer method despite possible underestimation of NFR. Our results showed that >10 μm fraction accounted for 60% of bulk NFR. Measurable NFR was detected in the CE controlled by the CDW where was characterized by nitrogen repletion and low-density of *Trichodesmium*. In addition, abundant *Trichodesmium* and active N_2_ fixation were observed in the southeastern ECS because of the Kuroshio intrusion. We hypothesize that the present NFRs of >10 μm and <10 μm fractions were contributed by *Trichodesmium* and proteobacteria, respectively. We concluded that spatial distribution of NFR off the CE was largely regulated by water mass (intruded Kuroshio water and CDW) movement and associated diazotrophs (particularly *Trichodesmium*) and nutrient conditions. However, further studies need to be performed on diazotrophic composition using molecular sequencing and N_2_ fixation using the ^15^N_2_ dissolution tracer assay.

## Data availability statement

The raw data supporting the conclusions of this article will be made available by the authors, without undue reservation.

## Author contributions

ZJ collected and analyzed the samples, developed the method, and wrote, reviewed, and edited the original draft of this manuscript. YZ analyzed the NFR data and conducted statistical test. ZS was responsible for the figure depiction and partial statistical analysis. HZ helped to perform the NFR incubation experiment and provided the chlorophyll *a* data. FZ provided the physical data. XY and QC developed the research design and revised the manuscript. JC provided nutrient data. JZ reviewed and edited this manuscript. All authors contributed to the article and approved the submitted version.

## Funding

This work was funded by the National Natural Science Foundation of China (41876198 and 42076134), Scientific Research Fund of the Second Institute of Oceanography, MNR (JG2311), Zhejiang Provincial Natural Science Foundation of China (LR22D060001), National Key Research and Development Program of China (2021YFC3101702), Key R&D Program of Zhejiang (2022C03044), and Project of Long-term Observation and Research Plan in the Changjiang Estuary and Adjacent East China Sea (LORCE; SZ2001).

## Conflict of interest

The authors declare that the research was conducted in the absence of any commercial or financial relationships that could be construed as a potential conflict of interest.

## Publisher’s note

All claims expressed in this article are solely those of the authors and do not necessarily represent those of their affiliated organizations, or those of the publisher, the editors and the reviewers. Any product that may be evaluated in this article, or claim that may be made by its manufacturer, is not guaranteed or endorsed by the publisher.
